# Supplementary material for: Structural insights on ligand recognition at the human leukotriene B4 receptor 1
Source: Nat Commun. 2021 May 20;12:2971. doi: 10.1038/s41467-021-23149-1 (PMC8137929; doi:10.1038/s41467-021-23149-1)
Supplement: Supplementary file 1 — Supplementary Information [file 41467_2021_23149_MOESM1_ESM.pdf]

SUPPPLEMENTARY INFORMATION

Structural Insights on Ligand Recognition at the Human Leukotriene B4 Receptor 1

Nairie Michaelian[1,2], Anastasiia Sadybekov[1,2], Élie Besserer-Offroy[3,4], Gye Won Han[1,2], Harini Krishnamurthy[5], Beata A. Zamlynny[5], Xavier Fradera[5], Phieng Siliphaivanh[5], Jeremy Presland[5], Kerrie B. Spencer[5], Stephen M. Soisson[5], Petr Popov[6,7], Philippe Sarret[3], Vsevolod Katritch[1,2,8], Vadim Cherezov[1,2,7]*

Author Affiliations

[1]Bridge Institute, USC Michelson Center for Convergent Bioscience, University of Southern California, Los Angeles, CA 90089, USA.

[2]Department of Chemistry, University of Southern California, Los Angeles, CA 90089, USA.

[3]Department of Pharmacology-Physiology, Faculty of Medicine and Health Sciences, Institut de Pharmacologie de Sherbrooke, Université de Sherbrooke, Sherbrooke, Québec, Canada.

[4]Department of Molecular and Medical Pharmacology, David Geffen School of Medicine, University of California at Los Angeles, Los Angeles, CA 90095, USA.

[5]Merck Research Laboratories, Merck & Co., Inc., Kenilworth, NJ 07033, USA.

[6]Center for Computational and Data Intensive Science and Engineering, Skolkovo Institute of Science and Technology, Moscow 121205, Russia.

[7]Research Center for Molecular Mechanisms of Aging and Age-Related Diseases, Moscow Institute of Physics and Technology, Dolgoprudny 141701, Russia.

[8]Department of Quantitative and Computational Biology, University of Southern California, Los Angeles, CA 90089, USA.

*Correspondence to Vadim Cherezov (cherezov@usc.edu)

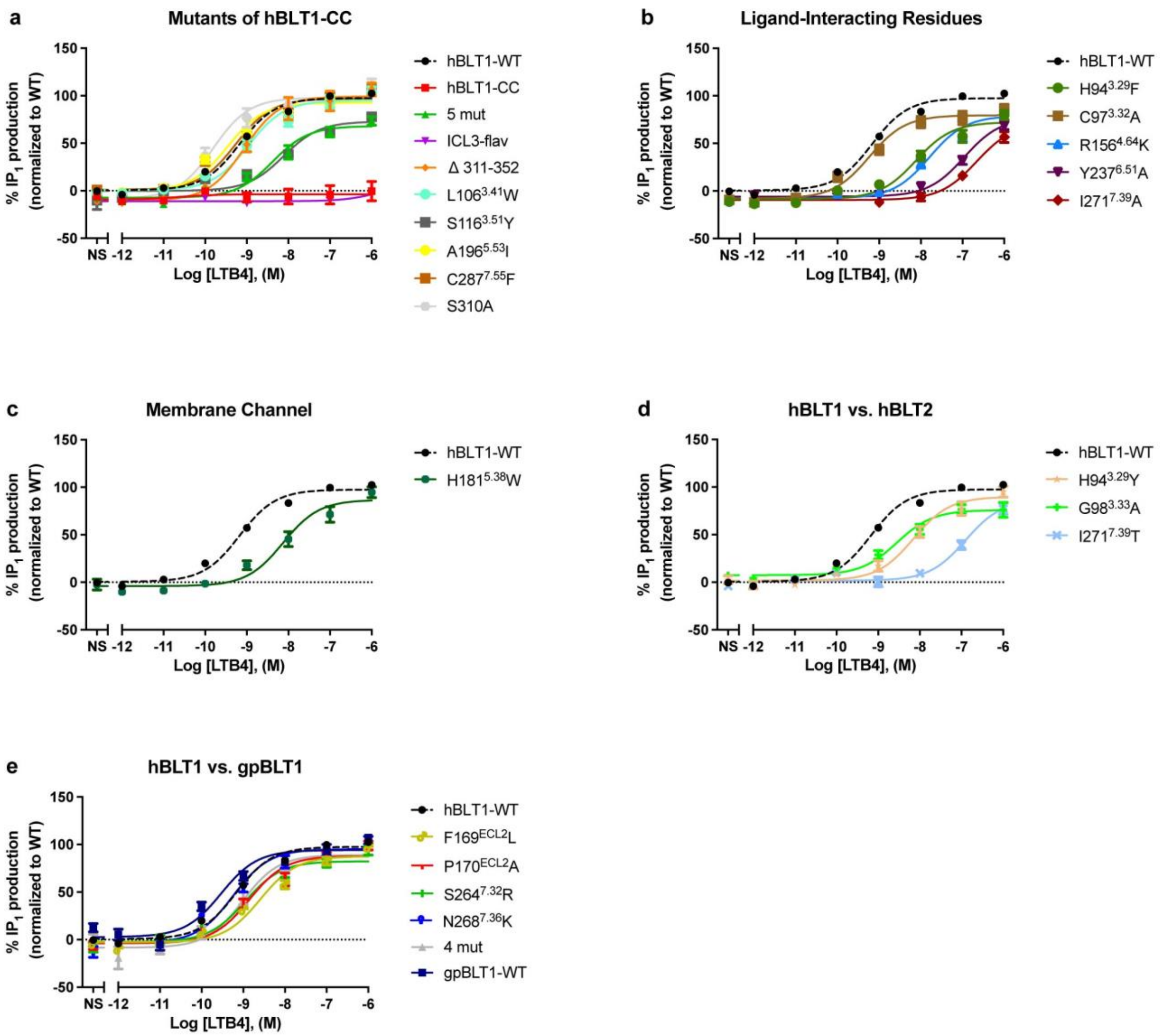

Supplementary Fig. 1: Signaling data for hBLT1 mutants induced by LTB4.

a-e Results are expressed as mean % of IP$_1$ production ± SEM from at least three independent experiments carried out in quadruplicate. Symbols in figure: hBLT1 or hBLT2, human leukotriene B4 receptor 1 or 2; gpBLT1, guinea pig BLT1; IP$_1$, myo-inositol 1 phosphate; LTB4, leukotriene B4; WT, wild type; CC, crystallization construct; 5 mut, 5 mutations from hBLT1-CC (L106$^{3.41}$W, S116$^{3.51}$Y, A196$^{5.53}$I, C287$^{7.55}$F, and S310A); ICL3-flav, ICL3-flavodoxin; Δ 311-352, truncation of hBLT1 residues 311-352; 4 mut, 4 non-conserved residues in the hBLT1 binding pocket mutated to their gpBLT1 equivalents (F169^{ECL2}L, P170^{ECL2}A, S264$^{7.32}$R, N268$^{7.36}$K).

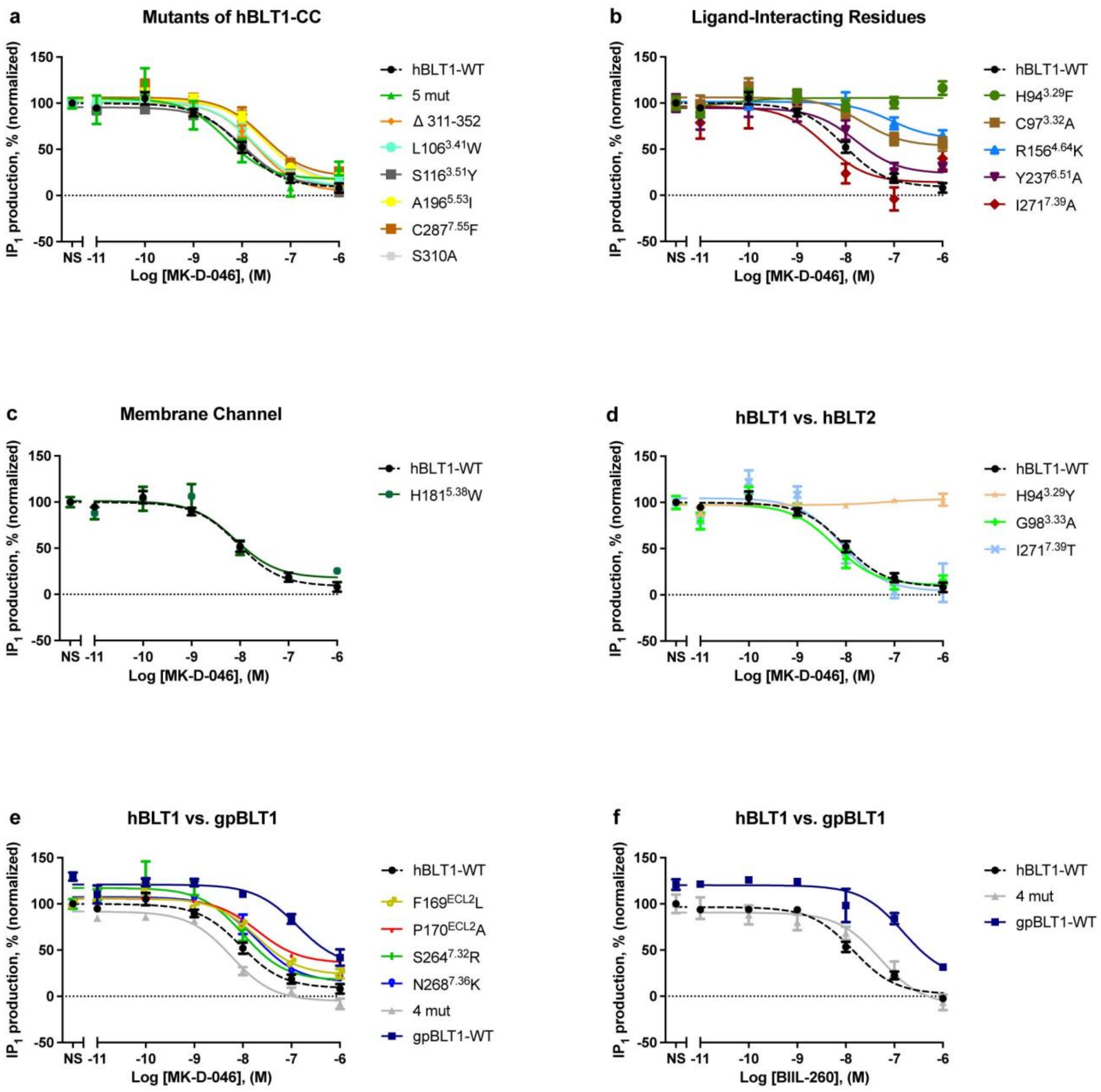

Supplementary Fig. 2: Inhibition of LTB4-induced signaling for hBLT1 mutants by MK-D-046 or BIIL-260. **a-f** Results are expressed as mean % of IP_1 production ± SEM from three independent experiments carried out in quadruplicate. Symbols in figure: hBLT1 or hBLT2, human leukotriene B4 receptor 1 or 2; gpBLT1, guinea pig BLT1; IP_1, myo-inositol 1 phosphate; LTB4, leukotriene B4; WT, wild type; CC, crystallization construct; 5 mut, 5 mutations from hBLT1-CC (L106$^{3.41}$W, S116$^{3.51}$Y, A196$^{5.53}$I, C287$^{7.55}$F, and S310A); ICL3-flav, ICL3-flavodoxin; Δ 311-352, truncation of hBLT1 residues 311-352; 4 mut, 4 non-conserved residues in the hBLT1 binding pocket mutated to their gpBLT1 equivalents (F169^{ECL2}L, P170^{ECL2}A, S264$^{7.32}$R, N268$^{7.36}$K).

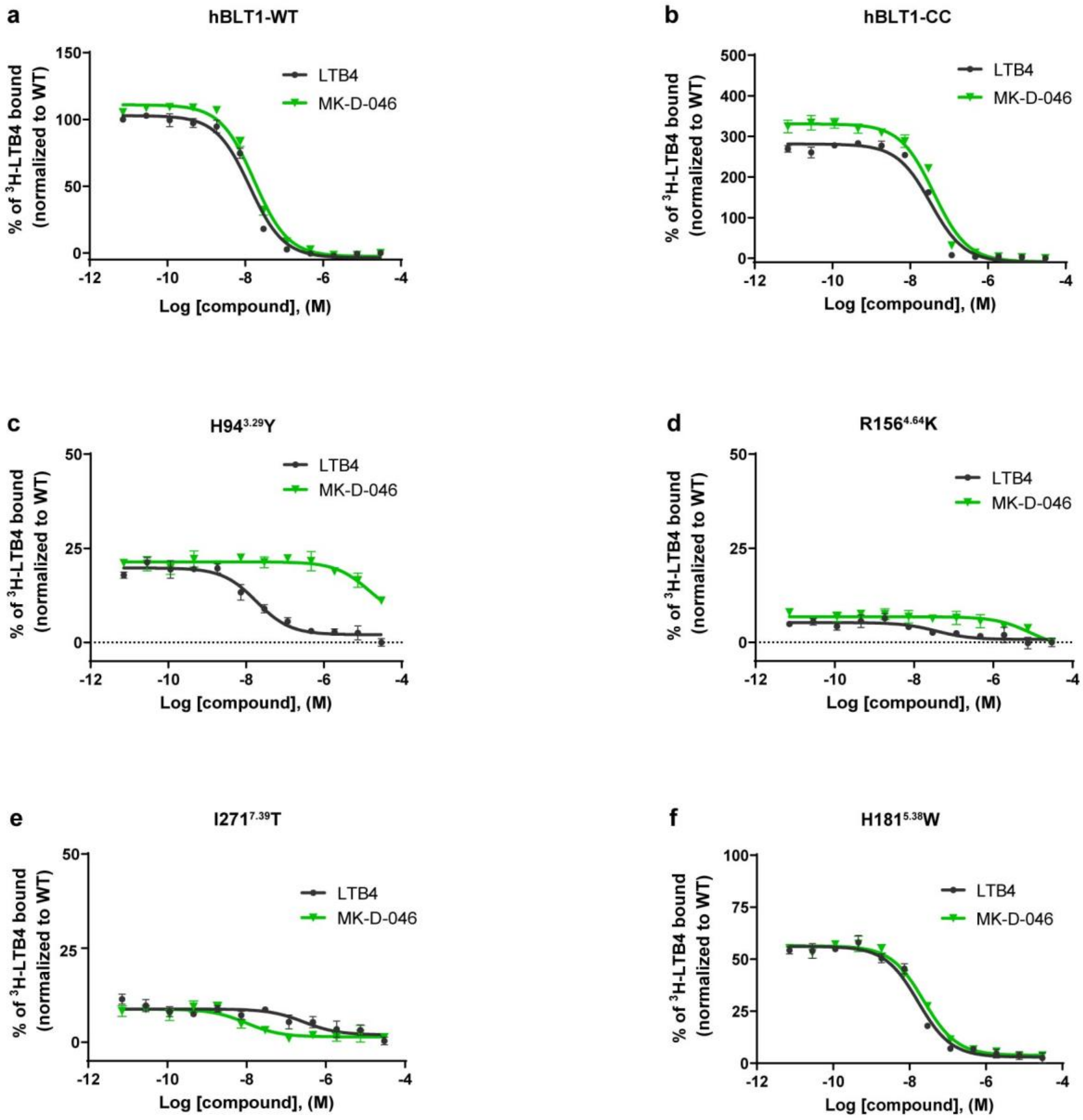

Supplementary Fig. 3: Radioligand [^{3}H]–LTB$_4$ competition binding for LTB4 and MK-D-046.

a-f Results are expressed as mean % of [^{3}H]–LTB4 bound normalized to WT ± SEM for n = 3 independent experiments. Membrane preparations of *Sf9* insect cells expressing hBLT1-WT or mutants were incubated with 4 nM [^{3}H]–LTB$_4$, and specific binding was measured in the presence of the indicated concentrations of LTB4 or MK-D-046. Symbols in figure: hBLT1, human leukotriene B4 receptor 1; LTB4, leukotriene B4; WT, wild type; CC, crystallization construct.

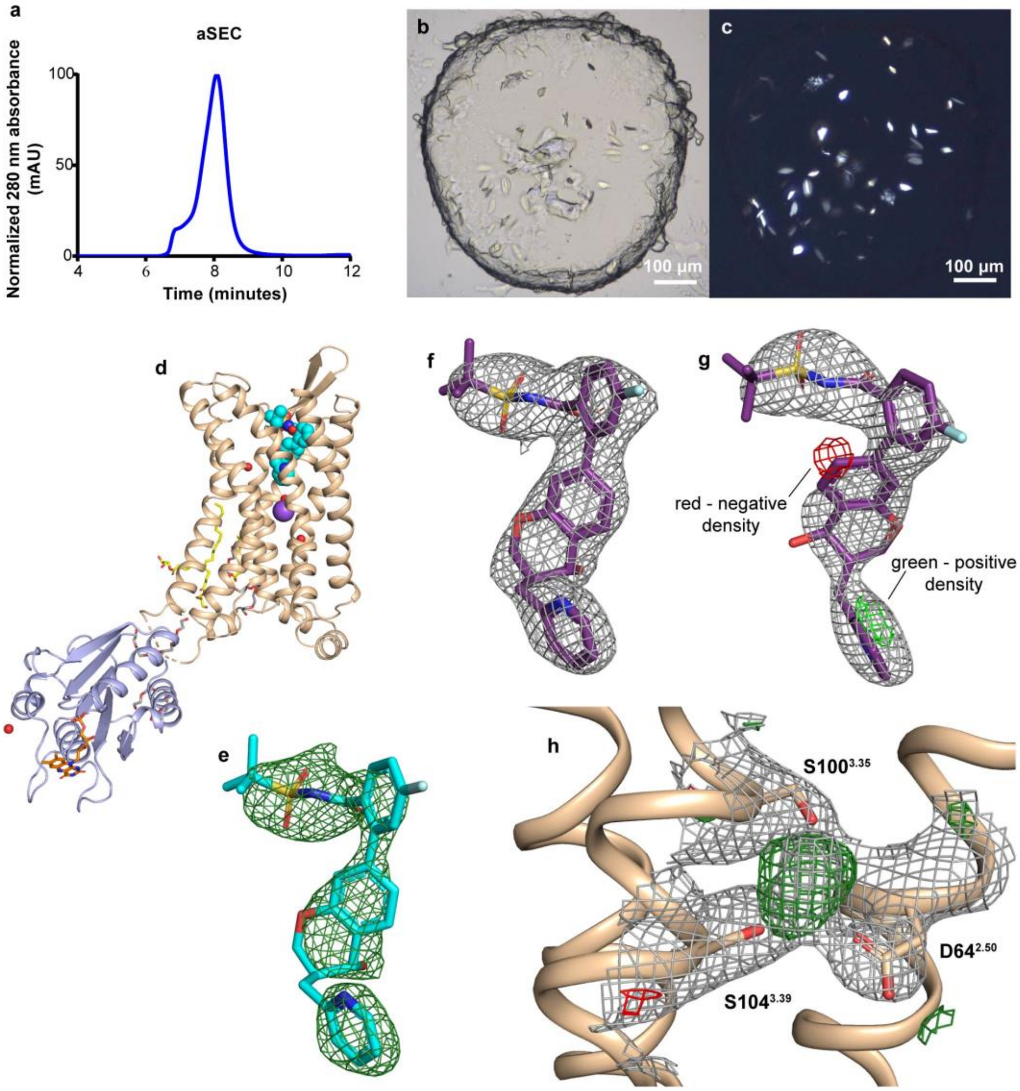

Supplementary Fig. 4: Crystallization and structure determination of hBLT1 in complex with MK-D-046. **a** Representative aSEC profile of the hBLT1 crystallization construct (hBLT1-CC) co-purified with MK-D-046. **b, c** Bright field (**b**) and cross-polarized (**c**) images of crystals of hBLT1-CC in complex with MK-D-046 used for crystallographic data collection. **d** Overall structure of hBLT1-CC in complex with MK-D-046. hBLT1 and artificial sequence are shown in wheat and flavodoxin is shown in violet. MK-D-046 (cyan), sodium (Na^+, purple) and water (red) are depicted as spheres. FMN (orange), lipids (yellow), and PEG molecules (grey) are depicted as sticks. **e** Omit mF_o-DF_c density (green mesh) of MK-D-046 contoured at 3.0 σ. **f, g** MK-D-046 can be modeled in 2 conformations (**f** and **g**) with alternate orientations of the chromanol core. Refined $2mF_o$-DF_c density (grey mesh) is contoured at 1.0 σ and refined mF_o-DF_c density (green – positive, red – negative) is contoured at ± 3.0 σ. Both conformations were refined in parallel and the conformation shown in (**f**) was used in the final crystal structure of hBLT1 due to a better ligand fit and an absence of strong mF_o-DF_c densities at ± 3.0 σ. **h** Omit electron density inside the sodium pocket in hBLT1. Omit $2mF_o$-DF_c electron density (grey mesh) is contoured at 1.0 σ and omit mF_o-DF_c density (green – positive, red – negative) is contoured at ± 3.0 σ.

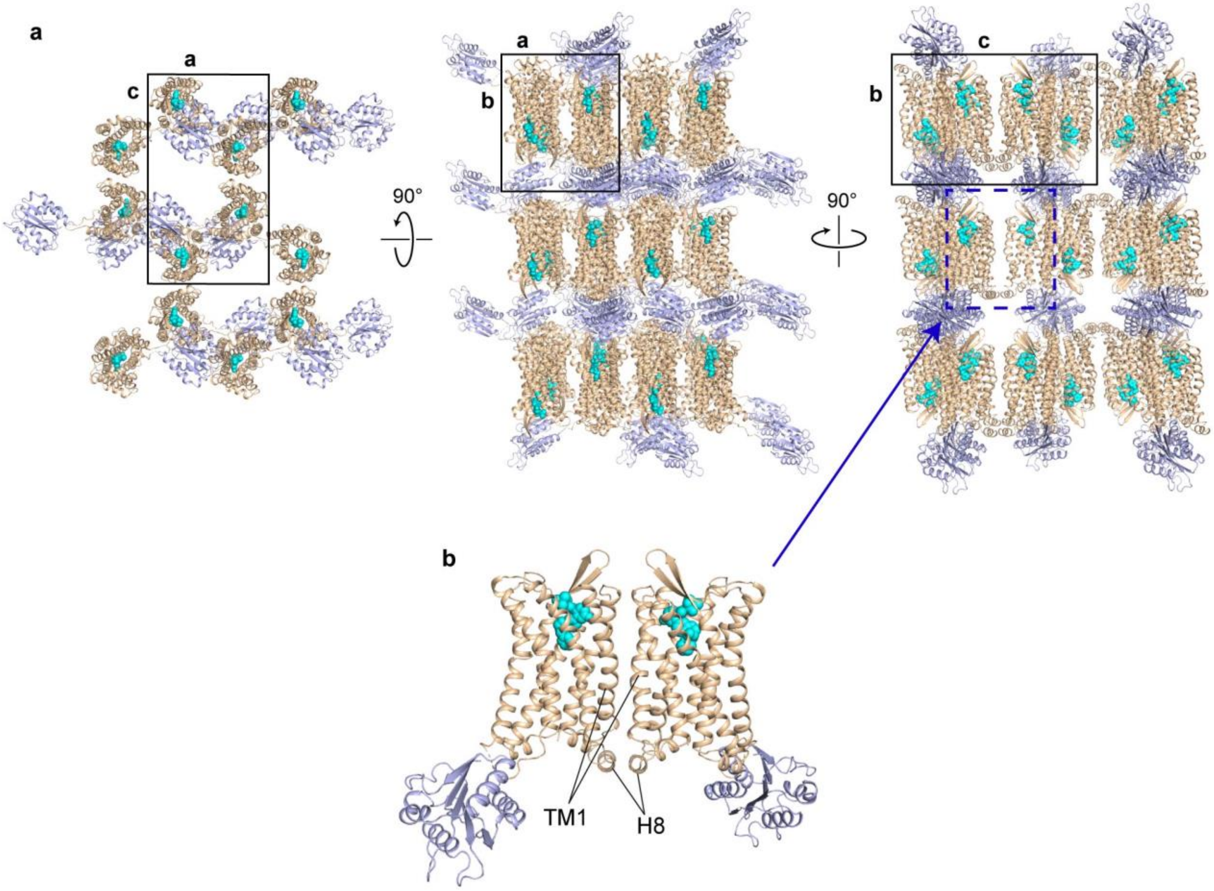

Supplementary Fig. 5: Crystal packing of hBLT1 in complex with MK-D-046.

a Overall crystal packing of the hBLT1 crystallization construct (hBLT1-CC) in complex with MK-D-046 (cyan spheres). hBLT1 is shown in wheat and flavodoxin is shown in violet. Receptors are arranged in layers with ICL3-flavodoxin mediating most of the hydrophilic contacts. Top view (left panel) only shows a single layer. Unit cell is shown as a black box and a, b, and c axes are labeled accordingly. **b** Crystallographic parallel dimer of hBLT1-CC in complex with MK-D-046. Location of parallel packing in overall crystal packing is outlined in a blue dashed line in the right panel of (**a**). Symbols in figure: TM1, transmembrane helix 1; H8, helix 8.

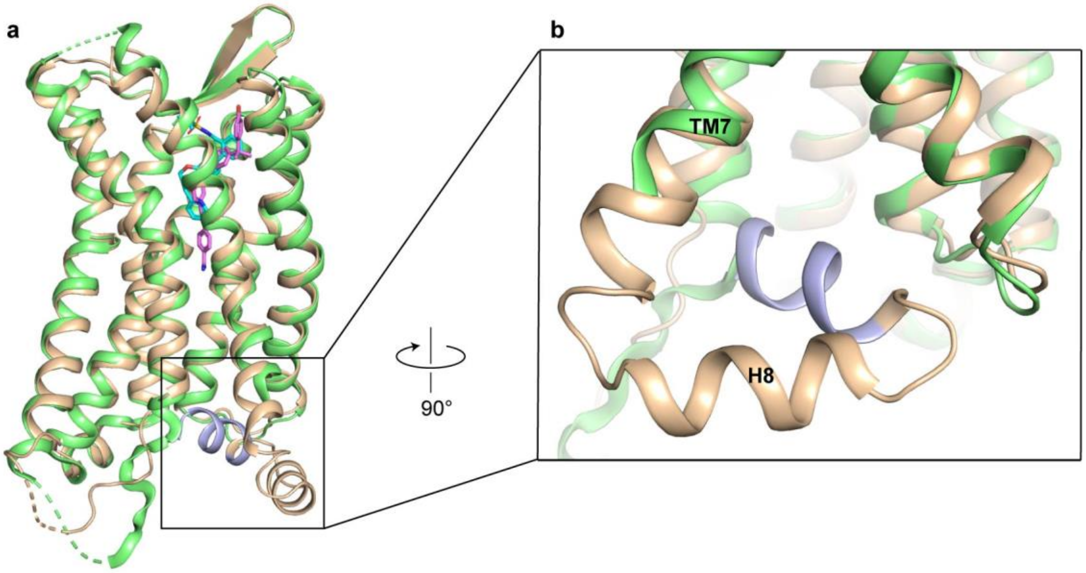

Supplementary Fig. 6: hBLT1 has a fully resolved TM7 and H8.
a Overlay of the hBLT1 (wheat) and gpBLT1 (PDB ID 5X33, green) structures. **b** Close-up of the intracellular end of TM7 and H8 that are fully resolved in hBLT1 but not in gpBLT1**.** Portion corresponding to the artificial sequence, or the EcoRI site + PreScission Protease recognition site, is shown in violet. Symbols in figure: TM7, transmembrane helix 7; H8, helix 8.

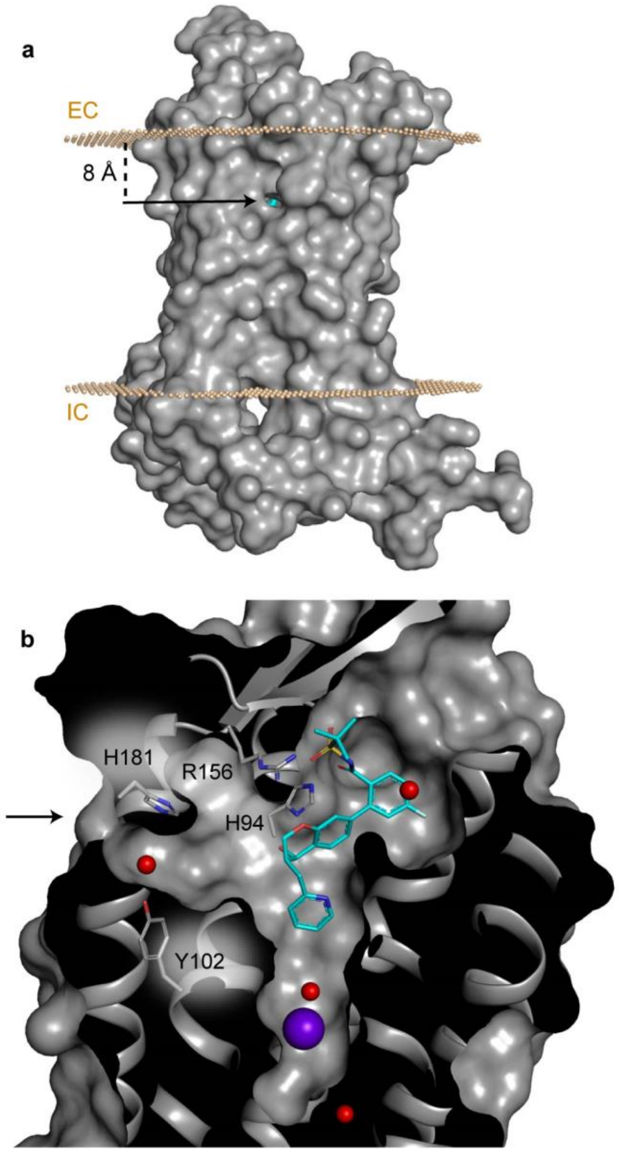

Supplementary Fig. 7: Putative membrane channel in hBLT1.

a Surface representation of the hBLT1 structure showing an opening into the membrane channel (horizontal black arrow) with a view of MK-D-046 (cyan). The membrane boundaries (wheat spheres; EC, extracellular side; IC, intracellular side) were obtained from the Orientations of Proteins in Membranes (OPM) database[1] and are the same as those shown in Fig. 1a. The distance from the EC membrane boundary to the center of the membrane channel (dashed black line) is ~8 Å. **b** Cross-section of the hBLT1 binding pocket with the membrane channel. A few residues are shown for reference. Black arrow indicates entry of membrane channel as shown in **a**. MK-D-046 is shown as sticks with cyan carbons. Sodium (Na^+, purple) and water (red) are shown as spheres. **c, d** Two possible conformations of LTB4 (sticks with green or yellow carbons) binding in cross-sections of hBLT1, with residues that have been mutated in functional studies here and in other studies. Conformation 1 (**c**) extends into the membrane channel and conformation 2 (**d**) extends down towards the sodium site. Conformation 1 and 2 are shown with the all-trans conformation of the triene of LTB4.

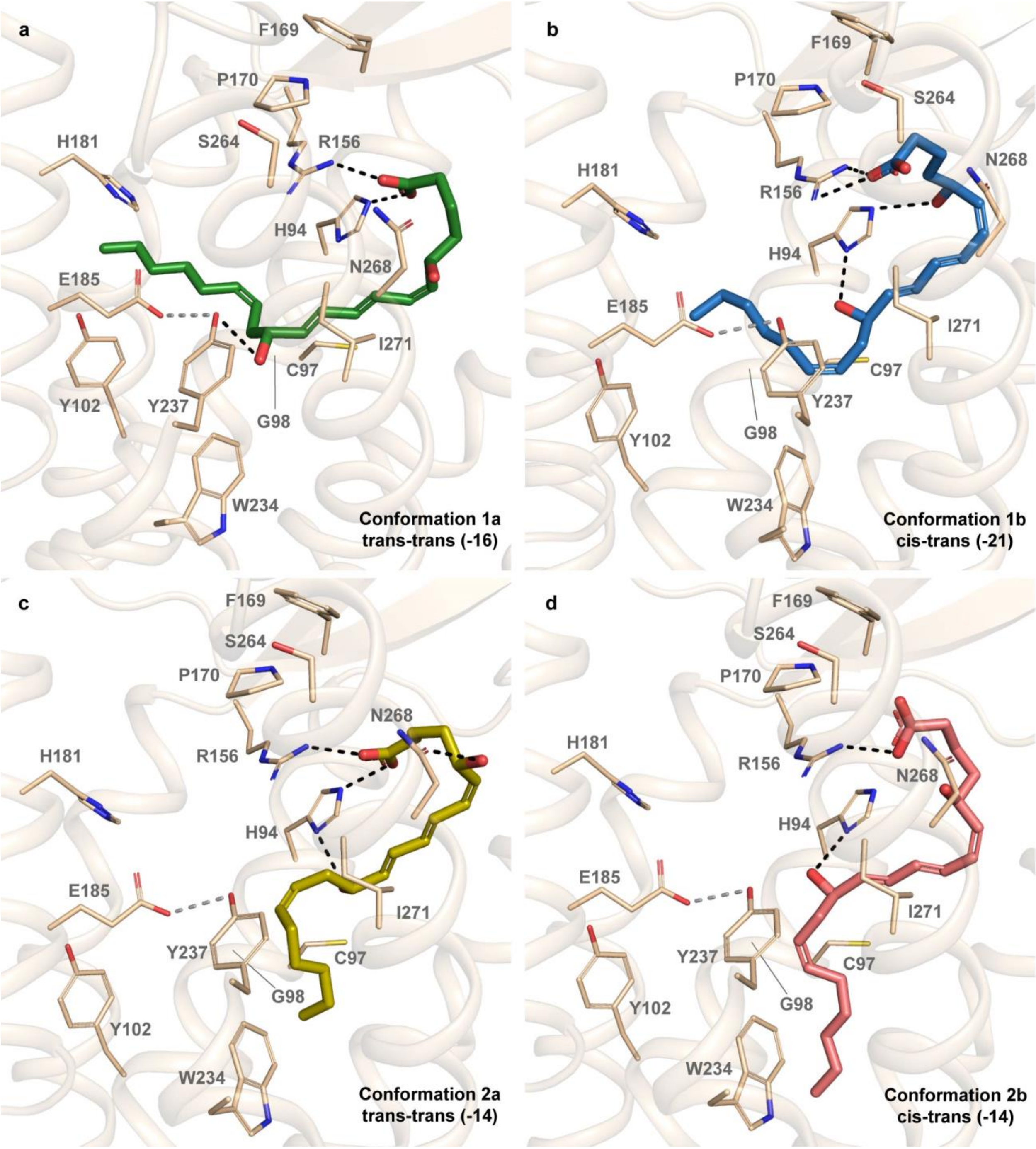

Supplementary Fig. 8: Docking of LTB4 in hBLT1.

Docking revealed four possible conformations of LTB4 in hBLT1. **a, b** Conformation 1 with the triene group in all-trans (1a) and cis-trans (1b) conformations. **c, d** Conformation 2 with the triene group in all-trans (2a) and cis-trans (2b) conformations. Docking scores are noted for each conformation in parentheses. Residues represented as sticks were evaluated in functional assays here and in other studies. Hydrogen bonds and salt bridges are represented as dashed lines.

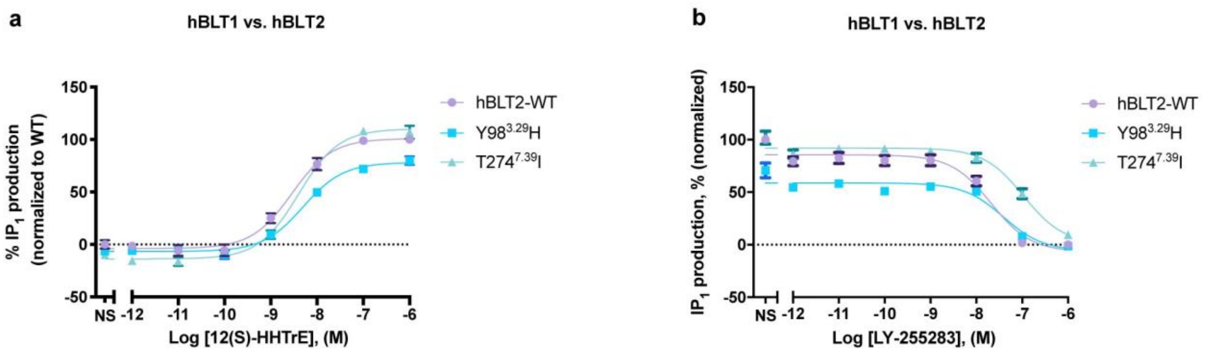

Supplementary Fig. 9: Signaling data for hBLT2 mutants induced by 12(S)-HHTrE and inhibition of signaling by LY-255283.

a-b Results for stimulation of IP_1 production by the agonist 12(S)-HHTrE (**a**) and inhibition of 12(S)-HHTrE-induced IP_1 production by the antagonist LY-255283 (**b**) are expressed as mean % of IP_1 production ± SEM from n = 3 independent experiments carried out in quadruplicate. Symbols in figure: hBLT1 or hBLT2, human leukotriene B4 receptor 1 or 2; IP_1, myo-inositol 1 phosphate; 12(S)-HHTrE, 12-hydroxyheptadecatrienoic acid; WT, wild type.

Supplementary Table 1: Binding data for hBLT1-WT and mutants evaluated in competition radioligand binding assays with ^{3}H-LTB4.

Category	Mutation	K_i LTB4, nM ± SEM	B_{max}, % ± SEM	K_i MK-D-046, nM ± SEM	B_{max}, % ± SEM
**Wild Type**	hBLT1-WT	3.4 ± 1.1	100 ± 2	4.4 ± 1.1	105 ± 3
**hBLT1-CC**	hBLT1-CC	8.8 ± 1.2	270 ± 8	11.0 ± 1.2	324 ± 16
**Ligand-Interacting Residues / hBLT1 vs. hBLT2**	H94$^{3.29}$Y	4.8 ± 1.3	17.9 ± 0.8	3,800 ± 300	21.1 ± 0.6
	R156$^{4.64}$K	9.5 ± 2.4	4.7 ± 0.7	2,200 ± 170	8 ± 1
	I271$^{7.39}$T	73 ± 2	11.4 ± 1.4	2.6 ± 1.8	8.3 ± 1.4
**Membrane Channel**	H181$^{5.38}$W	4.4 ± 1.0	54.3 ± 1.7	6.1 ± 1.0	55.4 ± 1.1

Results are expressed as mean ± SEM from n = 3 independent experiments. Symbols in table: hBLT1 or hBLT2, human leukotriene B4 receptor 1 or 2; LTB4, leukotriene B4; WT, wild type; $K_{i,}$ dissociation constant determined from competition binding assays; B_{max}, maximum number of binding sites in comparison to hBLT1-WT; CC, crystallization construct.

Supplementary Table 2: Crystallographic data collection and refinement statistics.

	hBLT1 – MK-D-046
**Data collection**	
Space group	$P2_1\ 2\ 2_1$
Cell dimensions	
a, b, c (Å)	70.77, 82.67, 127.48
Resolution (Å)	40.0 – 2.9 (3.0 – 2.9)
R_{sym} or R_{merge}	0.246 (1.83)
I / σI	7.48 (0.60)
$CC_{1/2}$	0.985 (0.314)
Completeness (%)	84.9 (50.0)#
Redundancy	8.9 (4.1)
**Refinement**	
Resolution (Å)	34.4 – 2.9 (2.9, 2.9, 3.6) ‡
No. reflections	13,808
R_{work} / R_{free}	0.212 / 0.261
No. atoms	
Protein	3,439
Ligand	35
Na^+ ion	1
Lipid and other	149
B-factors (Å^2)	
Protein	63.4
Ligand	72.1
Na^+ ion	67.2
Lipid and other	65.0
R.m.s. deviations	
Bond lengths (Å)	0.003
Bond angles (°)	0.55

Values inside parentheses correspond to the highest-resolution shell. The number of crystals used is 32.

#The completeness of the highest resolution shell is low due to the anisotropic diffraction. Data collection statistics are shown before anisotropic truncation.

‡Resolution limits along the a*, b*, and c* axes as defined by the STARANISO server.[2]

Supplementary Table 3: Contact area and percent of buried surface area of hBLT1 residues that interact with MK-D-046.

Residue	Contact Area (Å^2)	Percent Buried (%)
I271$^{7.39}$	41.8	34
H94$^{3.29}$	36.5	29
C97$^{3.32}$	30.0	31
R156$^{4.64}$	26.3	16
F74$^{2.60}$	25.2	16
F275$^{7.43}$	24.1	18
W234$^{6.48}$	16.2	8

Ligand-interacting residues with a contact area of less than 15 Å^2 are not included.

Supplementary Table 4: Cell surface expression and signaling data for hBLT1 mutants evaluated in IP_1 production assays with BIIL-260.

Category	Mutation	Cell surface expression, % of WT ± SEM	IC_{50} BIIL-260, nM ± SEM	I_{max}, % ± SEM
**Wild Type**	hBLT1-WT	100 ± 8 (5)	37 ± 8 (5)	100 ± 5 (5)
**hBLT1 vs. gpBLT1**	4 mut	84 ± 15 (3)	51 ± 16 (3)	107 ± 6 (3)
	gpBLT1-WT	60 ± 8 (3)	158 ± 12 (5)	68 ± 6 (5)

Results are expressed as mean ± SEM from at least three independent experiments carried out in triplicate (cell surface expression data) or quadruplicate (signaling data). The number of independent experiments (n) is shown in parenthesis. Cell surface expression of mutants are reported as % of hBLT1-WT. Symbols in table: hBLT1 or gpBLT1, human or guinea pig leukotriene B4 receptor 1; IP_1, myo-inositol 1 phosphate; WT, wild type; IC_{50} and I_{max}, potency and efficacy of BIIL-260 inhibition of IP_1 production; 4 mut, 4 non-conserved residues in the hBLT1 binding pocket mutated to their gpBLT1 equivalents ($F169^{ECL2}L$, $P170^{ECL2}A$, $S264^{7.32}R$, $N268^{7.36}K$). Expression data for constructs are shown here for reference and are the same as those shown in Table 1.

Supplementary Table 5: Docking comparisons between hBLT1 and gpBLT1.

Name	Chemical Structure	hBLT1 docking results	gpBLT1 docking results
1. MK-D-046	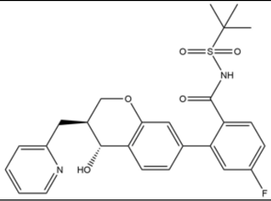	-29<br>consistent	-10<br>not consistent
2. CP-195,543	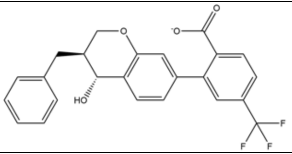	-29<br>consistent	-21<br>not consistent
3. BIIL-260	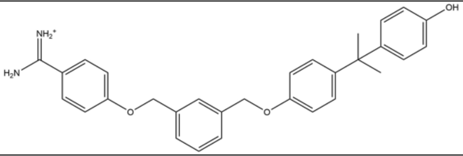	-38<br>consistent	-33<br>consistent
4. CGS-25019C	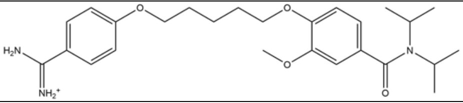	-31<br>consistent	-18<br>consistent
5. DW-1350	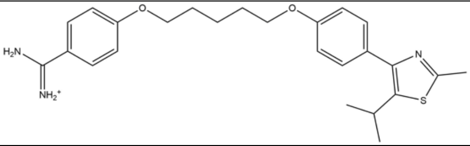	-34<br>consistent	-25<br>consistent
6. LY-293111	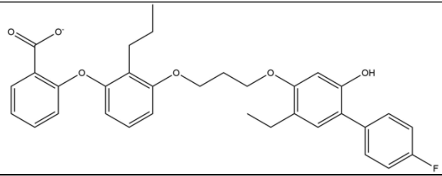	-13<br>consistent	-11<br>consistent
7. ONO-4057	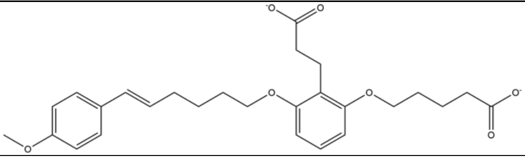	-30<br>consistent	-25<br>consistent
8. Structure 33*	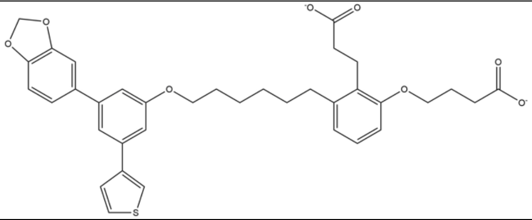	-17<br>consistent	-15<br>not consistent

Results from docking studies comparing hBLT1 and gpBLT1 (PDB ID 5X33) structures. 'Consistent' indicates that the ligand has a conformation similar to either MK-D-046 or BIIL-260 from the crystal structures, and important interactions are preserved. 'Not consistent' indicates that the ligand has one or more of the following characteristics: the pose is different from both MK-D-046 or BIIL-260 in the crystal structures, important interactions are not preserved, or the ligand is docked outside the binding pocket. *Preclinical compound from Supplementary Ref. 3.

Supplementary Table 6: Structure-activity relationship (SAR) analysis of MK-D-046 and its analogs.

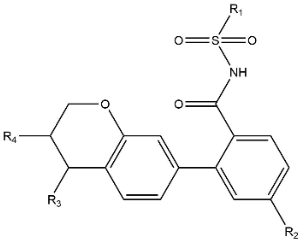

Example[4]	R_1	R_2	R_3	R_4	IC_{50} (nM)	hBLT1 docking score	Effects of substituents
**14 (MK-D-046)**	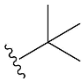	F	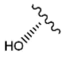	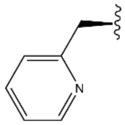	2	-29	Tert-butyl anchors the carbonyl sulfonamide for polar interactions with $H94^{3.29}$ and $R156^{4.64}$; fluorine interacts with a hydrophobic sub-pocket; hydroxyl forms a hydrogen bond with $H94^{3.29}$; pyridine interacts with the bulky residues in the lower part of the binding pocket.
**4**	CH_3	F	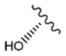	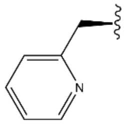	93	-26	Replacing tert-butyl with a smaller methyl group causes the carbonyl sulfonamide to rotate and results in less optimal hydrogen bonds with $R156^{4.64}$.
**12**	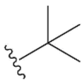	H	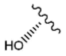	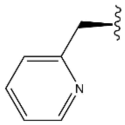	17	-29	Loss of fluorine reduces interactions with the hydrophobic sub-pocket.
**13**	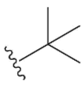	F	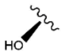	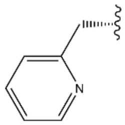	441	-26	Chiral opposite of the hydroxyl group causes a shift of the chromanol core to maintain the hydrogen bond with $H94^{3.29}$.
**16**	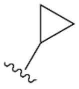	F	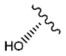	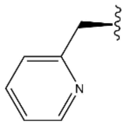	4	-25	Cyclopropane anchors the carbonyl sulfonamide similar to tert-butyl and maintains similar hydrogen bonds with $H94^{3.29}$ and $R156^{4.64}$; therefore, there is little change in potency.
**37**	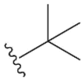	H	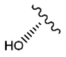	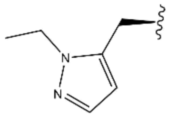	36	-14	Loss of fluorine reduces interactions with the hydrophobic sub-pocket; the ethyl-diazole group is larger than pyridine and clashes with bulky residues in the lower part of the binding pocket.

SAR and docking results for MK-D-046 and its analogs. Potency data (IC_{50}) are obtained from Supplementary Ref. 4. All ligands from this series are docked in a consistent conformation similar to MK-D-046 from the hBLT1 crystal structure, and important interactions are preserved.

Supplementary Table 7: Cell surface expression and signaling data for hBLT2 mutants evaluated in IP_1 production assays.

Category	Mutation	Cell surface expression, % of WT ± SEM[a]	EC_{50} 12(S)-HHTrE, nM ± SEM[b]	E_{max}, % ± SEM[b]	IC_{50} LY-255283, nM ± SEM[b]	I_{max}, % ± SEM[b]
**Wild Type**	hBLT2-WT	100 ± 3	2.8 ± 0.6	100 ± 4	21 ± 5	100 ± 2
**hBLT1 vs. hBLT2**	$Y98^{3.29}H$	80 ± 6	5.0 ± 0.9	80 ± 4	39 ± 7	101 ± 2
	$T274^{7.39}I$	109 ± 3	4.0 ± 0.6	107 ± 6	107 ± 16	91 ± 3

Results are expressed as mean ± SEM from n = 3 independent experiments carried out in triplicate (cell surface expression data) or quadruplicate (signaling data). Cell surface expression of mutants are reported as % of hBLT2-WT. Symbols in table: hBLT1 or hBLT2, human leukotriene B4 receptor 1 or 2; IP_1, myo-inositol 1 phosphate; 12(S)-HHTrE, 12-hydroxyheptadecatrienoic acid; WT, wild type; EC_{50} and E_{max}, potency and efficacy of 12(S)-HHTrE; IC_{50} and I_{max}, potency and efficacy of LY-255283 inhibition of IP_1 production.

Supplementary Table 8: Primers used to generate constructs for hBLT1 and hBLT2 studies.

	**Mutation**	**Forward Primer (5'-3')**	**Reverse Primer (5'-to-3')**
**Primers for hBLT1-CC (*Sf9*)**	L106$^{3.41}$W	GGTGTTTCGATGTACGCTTCCGTGTGGC<br>TGATCACTGCCATGAGCTTGGAT	ATCCAAGCTCATGGCAGTGATCAGCCAC<br>ACGGAAGCGTACATCGAAACACC
	S116$^{3.51}$Y	ATCACTGCCATGAGCTTGGATCGCTAC<br>CTGGCTGTGGCCCGTCCTTTCGTC	GACGAAAGGACGGGCCACAGCCAGGTA<br>GCGATCCAAGCTCATGGCAGTGAT
	A196$^{5.53}$I	ACAGGATTCCTGCTCCCATTCCTCATCG<br>TCGTTGCCAGCTACTCTGACATT	AATGTCAGAGTAGCTGGCAACGACGAT<br>GAGGAATGGGAGCAGGAATCCTGT
	C287$^{7.55}$F	TCTGTGAACCCAGTCCTGTACGCATTCG<br>CAGGTGGAGGATTGCTGAGATCA	TGATCTCAGCAATCCTCCACCTGCGAAT<br>GCGTACAGGACTGGGTTCACAGA
	S310A	GCGAAACTCTTGGAGGGTACAGGCGCT<br>GAATTCCTGGAGGTGCTCTTCCAG	CTGGAAGAGCACCTCCAGGAATTCAGCG<br>CCTGTACCCTCCAAGAGTTTCGC
	ICL3-flav	GGTAGGAGACTGCAGGCTCGCCGTGCC<br>AAGGCTCTCATCGTGTATGGA	ACGGCGAGATCTCCTGAAACGGCGAAT<br>AGCGCCCCTCACGTCATGGGC
**Primers for hBLT1-WT and mutant constructs tested in IP_1 Production Assays (HEK293)**	+ 3×HA tag	CACTAGTCCAGTGTGGTGGAATTCACC<br>ATGTACCCATACGATGTTCCAGATTAC<br>GCTTACCCATACGATGTTCCAGATTAC<br>GCTTACCCATACGATGTTCCAGATTAC<br>GCTGATAACACTACATCTTCTGCAGCA<br>CCCCCCTCA	TGAGGGGGGTGCTGCAGAAGATGTAGT<br>GTTATCAGCGTAATCTGGAACATCGTAT<br>GGGTAAGCGTAATCTGGAACATCGTATG<br>GGTAAGCGTAATCTGGAACATCGTATGG<br>GTACATGGTGAATTCCACCACACTGGAC<br>TAGTG
	Δ 311-352	GCCAAGCTGCTGGAGGGCACGGGCTCC<br>TAGCTCGAGTCTAGAGGGCCCGTTTAA	TTAAACGGGCCCTCTAGACTCGAGCTAG<br>GAGCCCGTGCCCTCCAGCAGCTTGGC
	L106$^{3.41}$W	CACTATGTCTGCGGAGTCAGCATGTAC<br>GCCAGCGTCTGGCTTATCACGGCCATG<br>AGTCTAGACCGCTCACTGGCG	CGCCAGTGAGCGGTCTAGACTCATGGCC<br>GTGATAAGCCAGACGCTGGCGTACATGC<br>TGACTCCGCAGACATAGTG
	S116$^{3.51}$Y	AGCGTCCTGCTTATCACGGCCATGAGT<br>CTAGACCGCTACCTGGCGGTGGCCCGC<br>CCCTTTGTGTCCCAGAAGCTA	TAGCTTCTGGGACACAAAGGGGCGGGC<br>CACCGCCAGGTAGCGGTCTAGACTCATG<br>GCCGTGATAAGCAGGACGCT
	A196$^{5.53}$I	TTCGAGGCTGTCACGGGCTTCCTGCTGC<br>CCTTCCTGATCGTGGTGGCCAGCTACTC<br>GGACATAGGGCGTCGGCTA	TAGCCGACGCCCTATGTCCGAGTAGCTG<br>GCCACCACGATCAGGAAGGGCAGCAGG<br>AAGCCCGTGACAGCCTCGAA
	C287$^{7.55}$F	TTCCTGAGCAGCAGCGTGAACCCCGTG<br>CTGTACGCGTTCGCCGGCGGCGGCCTG<br>CTGCGCTCGGCGGGCGTGGGC	GCCCACGCCCGCCGAGCGCAGCAGGCC<br>GCCGCCGGCGAACGCGTACAGCACGGG<br>GTTCACGCTGCTGCTCAGGAA
	S310A	GTGGGCTTCGTCGCCAAGCTGCTGGAG<br>GGCACGGGCGCCGAGGCGTCCAGCACG<br>CGCCGCGGGGGCAGCCTGGGC	GCCCAGGCTGCCCCCGCGGCGCGTGCTG<br>GACGCCTCGGCGCCCGTGCCCTCCAGCA<br>GCTTGGCGACGAAGCCCAC
	H94$^{3.29}$F	ACCTGGAGTTTTGGACTGGCTGGTTGC<br>CGCCTGTGTTTCTATGTCTGCGGAGTCA<br>GCATGTACGCCAGCGTCCTG	CAGGACGCTGGCGTACATGCTGACTCCG<br>CAGACATAGAAACACAGGCGGCAACCA<br>GCCAGTCCAAAACTCCAGGT
	C97$^{3.32}$A	TTTGGACTGGCTGGTTGCCGCCTGTGTC<br>ACTATGTCGCCGGAGTCAGCATGTACG<br>CCAGCGTCCTGCTTATCACG	CGTGATAAGCAGGACGCTGGCGTACATG<br>CTGACTCCGGCGACATAGTGACACAGGC<br>GGCAACCAGCCAGTCCAAA
	R156$^{4.64}$K	TTGTCCTTTCTGCTGGCCACACCCGTCC<br>TCGCGTACAAGACAGTAGTGCCCTGGA<br>AAACGAACATGAGCCTGTGC	GCACAGGCTCATGTTCGTTTTCCAGGGC<br>ACTACTGTCTTGTACGCGAGGACGGGTG<br>TGGCCAGCAGAAAGGACAA
	Y237$^{6.51}$A	CTCATCATCCTGACCTTCGCCGCCTTCT<br>GGCTGCCCGCCCACGTGGTGAACCTGG<br>CTGAGGCGGGCCGCGCGCTG	CAGCGCGCGGCCCGCCTCAGCCAGGTTC<br>ACCACGTGGGCGGGCAGCCAGAAGGCG<br>GCGAAGGTCAGGATGATGAG
	I271$^{7.39}$A	GTGGGGAAGCGGCTGAGCCTGGCCCGC<br>AACGTGCTCGCCGCACTCGCCTTCCTG<br>AGCAGCAGCGTGAACCCCGTG	CACGGGGTTCACGCTGCTGCTCAGGAAG<br>GCGAGTGCGGCGAGCACGTTGCGGGCC<br>AGGCTCAGCCGCTTCCCCAC
	H181$^{5.38}$W	TTCCCGCGGTACCCCAGCGAAGGGCAC<br>CGGGCCTTCTGGCTAATCTTCGAGGCT<br>GTCACGGGCTTCCTGCTGCCC	GGGCAGCAGGAAGCCCGTGACAGCCTC<br>GAAGATTAGCCAGAAGGCCCGGTGCCCT<br>TCGCTGGGGTACCGCGGGAA
	H94$^{3.29}$Y	ACCTGGAGTTTTGGACTGGCTGGTTGC<br>CGCCTGTGTTACTATGTCTGCGGAGTCA<br>GCATGTACGCCAGCGTCCTG	CAGGACGCTGGCGTACATGCTGACTCCG<br>CAGACATAGTAACACAGGCGGCAACCA<br>GCCAGTCCAAAACTCCAGGT
	G98$^{3.33}$A	GGACTGGCTGGTTGCCGCCTGTGTCAC<br>TATGTCTGCGCCGTCAGCATGTACGCC<br>AGCGTCCTGCTTATCACGGCC	GGCCGTGATAAGCAGGACGCTGGCGTA<br>CATGCTGACGGCGCAGACATAGTGACAC<br>AGGCGGCAACCAGCCAGTCC

Supplementary Table 8 continued

	Mutation	Forward Primer (5'-3')	Reverse Primer (5'-to-3')
**Primers for hBLT1-WT and mutant constructs in IP_1 Production Assays (HEK293)**	I271$^{7.39}$T	GTGGGGAAGCGGCTGAGCCTGGCCCGC<br>AACGTGCTCACCGCACTCGCCTTCCTG<br>AGCAGCAGCGTGAACCCCGTG	CACGGGGTTCACGCTGCTGCTCAGGAAG<br>GCGAGTGCGGTGAGCACGTTGCGGGCCA<br>GGCTCAGCCGCTTCCCCAC
	F169^{ECL2}L	CGCACAGTAGTGCCCTGGAAAACGAAC<br>ATGAGCCTGTGCCTGCCGCGGTACCCC<br>AGCGAAGGGCACCGGGCCTTCCATCTA	TAGATGGAAGGCCCGGTGCCCTTCGCTG<br>GGGTACCGCGGCAGGCACAGGCTCATGT<br>TCGTTTTCCAGGGCACTACTGTGCG
	P170^{ECL2}A	ACAGTAGTGCCCTGGAAAACGAACATG<br>AGCCTGTGCTTCGCCCCGGTACCCCAGC<br>GAAGGGCACCGGGCCTTCCATCTAATC	GATTAGATGGAAGGCCCGGTGCCCTTCG<br>CTGGGGTACCGGGCGAAGCACAGGCTCA<br>TGTTCGTTTTCCAGGGCACTACTGT
	S264$^{7.32}$R	GGCCAGGCCGCCGGGTTAGGGCTCGTG<br>GGGAAGCGGCTGCGGCTGGCCCGCAAC<br>GTGCTCATCGCACTCGCCTTCCTGAGC	GCTCAGGAAGGCGAGTGCGATGAGCAC<br>GTTGCGGGCCAGCCGCAGCCGCTTCCCC<br>ACGAGCCCTAACCCGGCGGCCTGGCC
	N268$^{7.36}$K	GGGTTAGGGCTCGTGGGGAAGCGGCTG<br>AGCCTGGCCCGCAAGGTGCTCATCGCA<br>CTCGCCTTCCTGAGCAGCAGCGTGAAC	GTTCACGCTGCTGCTCAGGAAGGCGAGT<br>GCGATGAGCACCTTGCGGGCCAGGCTCA<br>GCCGCTTCCCCACGAGCCCTAACCC
	4 mut, 1 of 2 (F169^{ECL2}L, P170 ^{ECL2}A)	TGGAAAACGAACATGAGCCTGTGCCTG<br>GCCCGGTACCCCAGCGAAGGGCACCGG	CCGGTGCCCTTCGCTGGGGTACCGGGCC<br>AGGCACAGGCTCATGTTCGTTTTCCA
	4 mut, 2 of 2 (S264$^{7.32}$R, N268$^{7.36}$K)	CTCGTGGGGAAGCGGCTGCGCCTGGCC<br>CGTAAAGTGCTCATCGCACTCGCCTTCC<br>TGAGC	GCTCAGGAAGGCGAGTGCGATGAGCACT<br>TTACGGGCCAGGCGCAGCCGCTTCCCCA<br>CGAG
**Primers for hBLT1 mutants in Radioligand Binding Assays (*Sf9*)** (primers for hBLT1-CC are listed above)	H94$^{3.29}$Y	GGCCTCGCTGGATGCAGGTTGTGTTATT<br>ACGTCTGCGGTGTTTCGATGTAC	GTACATCGAAACACCGCAGACGTAATAA<br>CACAACCTGCATCCAGCGAGGCC
	R156$^{4.64}$K	TTGGCTACGCCTGTGCTGGCCTACAAG<br>ACCGTTGTGCCCTGGAAGACTAAC	GTTAGTCTTCCAGGGCACAACGGTCTTG<br>TAGGCCAGCACAGGCGTAGCCAA
	I271$^{7.39}$T	TTGTCACTGGCTAGAAACGTTTTGACC<br>GCTCTGGCCTTCCTCTCCAGCTCT	AGAGCTGGAGAGGAAGGCCAGAGCGGT<br>CAAAACGTTTCTAGCCAGTGACAA
	H181$^{5.38}$W	CCGTCTGAGGGCCACAGAGCATTCTGG<br>TTGATCTTCGAAGCCGTCACAGGA	TCCTGTGACGGCTTCGAAGATCAACCAG<br>AATGCTCTGTGGCCCTCAGACGG
**Primers for hBLT2-WT and mutants in IP_1 Production Assays (HEK293)**	Y98$^{3.29}$H	GGCCAGGCCGGATGTAAAGCCGTGCAC<br>TACGTGTGCGCCCTGAGCATGTAC	GTACATGCTCAGGGCGCACACGTAGTGC<br>ACGGCTTTACATCCGGCCTGGCC
	T274$^{7.39}$I	GGACAGGCCGCCCGGGCCGGCACCATC<br>GCCCTCGCCTTTTTCAGCAGCAGC	GCTGCTGCTGAAAAAGGCGAGGGCGAT<br>GGTGCCGGCCCGGGCGGCCTGTCC

Primers were optimized for either insect cell (*Sf9*) or mammalian (HEK293) expression. Symbols in table: hBLT1 or hBLT2, human leukotriene B4 receptor 1 or 2; IP_1, myo-inositol 1 phosphate; WT, wild type; CC, crystallization construct; ICL3-flav, ICL3-flavodoxin; Δ 311-352, truncation of hBLT1 residues 311-352; + 3×HA tag, insertion of 3×hemagglutinin (HA) tag (YPYDVPDYA) at the N-terminus of hBLT1-WT and mutants; 4 mut, 4 non-conserved residues in the hBLT1 binding pocket mutated to their guinea pig BLT1 equivalents (F169^{ECL2}L, P170^{ECL2}A, S264$^{7.32}$R, N268$^{7.36}$K), F169^{ECL2}L and P170^{ECL2}A were combined in one primer and S264$^{7.32}$R and N268$^{7.36}$K were combined in another primer to obtain a total of 4 mutations in the final 4 mut construct.

Supplementary References

1. Lomize, M. A., Pogozheva, I. D., Joo, H., Mosberg, H. I. & Lomize, A. L. OPM database and PPM web server: resources for positioning of proteins in membranes. *Nucleic Acids Res* **40**, D370-376 (2012).

2. Tickle, I. J. *et al.* STARANISO. *Cambridge, United Kingdom: Global Phasing Ltd*, (2018).

3. Goodnow Jr., R. A. *et al.* Discovery of novel and potent leukotriene B4 receptor antagonists. Part 1. *J Med Chem* **53**, 3502-3516 (2010).

4. Han, Y., Lim, J., Siliphaivanh, P., Spencer, K. & Tummanapallli, S. Aryl Acylsulfonamides as BLT1 Antagonists. US Patent WO 2017/095722.
