# Supplementary material for: Structural insights on ligand recognition at the human leukotriene B4 receptor 1
Source: Nat Commun. 2021 May 20;12:2971. doi: 10.1038/s41467-021-23149-1 (PMC8137929; doi:10.1038/s41467-021-23149-1)
Supplement: Supplementary file 3 — Reporting Summary [file 41467_2021_23149_MOESM3_ESM.pdf]

nature research

Corresponding author(s): Vadim Cherezov

Last updated by author(s): Apr 12, 2021

Reporting Summary

Nature Research wishes to improve the reproducibility of the work that we publish. This form provides structure for consistency and transparency in reporting. For further information on Nature Research policies, see our Editorial Policies and the Editorial Policy Checklist.

Statistics

For all statistical analyses, confirm that the following items are present in the figure legend, table legend, main text, or Methods section.

n/a	Confirmed	
[ ]	[x]	The exact sample size (*n*) for each experimental group/condition, given as a discrete number and unit of measurement
[ ]	[x]	A statement on whether measurements were taken from distinct samples or whether the same sample was measured repeatedly
[x]	[ ]	The statistical test(s) used AND whether they are one- or two-sided<br>*Only common tests should be described solely by name; describe more complex techniques in the Methods section.*
[x]	[ ]	A description of all covariates tested
[x]	[ ]	A description of any assumptions or corrections, such as tests of normality and adjustment for multiple comparisons
[ ]	[x]	A full description of the statistical parameters including central tendency (e.g. means) or other basic estimates (e.g. regression coefficient) AND variation (e.g. standard deviation) or associated estimates of uncertainty (e.g. confidence intervals)
[x]	[ ]	For null hypothesis testing, the test statistic (e.g. *F*, *t*, *r*) with confidence intervals, effect sizes, degrees of freedom and *P* value noted<br>*Give P values as exact values whenever suitable.*
[x]	[ ]	For Bayesian analysis, information on the choice of priors and Markov chain Monte Carlo settings
[x]	[ ]	For hierarchical and complex designs, identification of the appropriate level for tests and full reporting of outcomes
[x]	[ ]	Estimates of effect sizes (e.g. Cohen's *d*, Pearson's *r*), indicating how they were calculated

Our web collection on statistics for biologists contains articles on many of the points above.

Software and code

Policy information about availability of computer code

Data collection	Crystallographic data were collected at the 23ID-B beamline of the Advanced Photon Source using JBluIce v. 2018.2 Build 6401
Data analysis	The following software was used in this study: HKL2000 v.718.05, Buster v. 2.10.2., Coot v. 0.8.9, PyMOL v. 2.3.3, Phenix v. 1.17.1-3660, ccp4 v. 7.0.078, ICM-Pro v. 3.8.7b, GraphPad Prism v. 9, STARANISO server v. 2.6.32 was accessed at http://staraniso.globalphasing.org on July 12 2019.

For manuscripts utilizing custom algorithms or software that are central to the research but not yet described in published literature, software must be made available to editors and reviewers. We strongly encourage code deposition in a community repository (e.g. GitHub). See the Nature Research guidelines for submitting code & software for further information.

Data

Policy information about availability of data

All manuscripts must include a data availability statement. This statement should provide the following information, where applicable:

- Accession codes, unique identifiers, or web links for publicly available datasets
- A list of figures that have associated raw data
- A description of any restrictions on data availability

Coordinates and structure factors for hBLT1 in complex with MK-D-046 have been deposited in the Protein Data Bank (PDB) with the accession code 7K15 (https://www.rcsb.org/structure/7K15). Raw diffraction images have been uploaded to Zenodo data repository with the DOI data identifier "10.5281/zenodo.4450301" (https://doi.org/10.5281/zenodo.4450301). The amino acid sequences for BLT1 and BLT2 receptors used in this study are available from the UniProt database under the accession numbers: Q15722 (hBLT1) [https://www.uniprot.org/uniprot/Q15722], Q9WTK1 (gpBLT1) [https://www.uniprot.org/uniprot/Q9WTK1], O88855 (mBLT1) [https://www.uniprot.org/uniprot/O88855], Q9R0Q2 (rBLT1) [https://www.uniprot.org/uniprot/Q9R0Q2], Q9NPC1 (hBLT2) [https://www.uniprot.org/uniprot/Q9NPC1]. All other data generated or analysed during this study are included in this published article and its supplementary information

files or are available from the corresponding author. Source data for Supplementary Figures 1, 2, 3, 9 are provided with the paper.

Field-specific reporting

Please select the one below that is the best fit for your research. If you are not sure, read the appropriate sections before making your selection.

☒ Life sciences ☐ Behavioural & social sciences ☐ Ecological, evolutionary & environmental sciences

For a reference copy of the document with all sections, see nature.com/documents/nr-reporting-summary-flat.pdf

Life sciences study design

All studies must disclose on these points even when the disclosure is negative.

Sample size	No statistical methods were used to predetermine sample size. IP1 signaling and ligand binding assays were conducted at least in n=3 biologically independent experiments and are comparable to other published studies (Zhang et al 2010 Assay Drug Dev Technol 8, 106-113; Maguire et al 2012 Methods Mol Biol 897, 31-77). Crystallographic data have been collected from 144 crystals. Data from 32 best diffracting crystals have been assembled together in a dataset with over 98% completeness and with redundancy above 10 in the lowest resolution shell.
Data exclusions	No data were excluded.
Replication	All pharmacological measurements were done at least in triplicates. All attempts at replication were successful. Few individual data points have been excluded as outliers based on the ROUT test with Q=0.1% in GraphPad Prism. Crystallization has been successfully repeated at least 5 times with reproducibly diffracting crystals.
Randomization	This study did not allocate experimental groups thus no randomization was required for the reported experiments.
Blinding	The researchers were not blinded to allocation during experiments and outcome assessment. Blinding was not required for the reported experiments because all functional and structural data were analyzed using the same methods, and results are not subjective.

Reporting for specific materials, systems and methods

We require information from authors about some types of materials, experimental systems and methods used in many studies. Here, indicate whether each material, system or method listed is relevant to your study. If you are not sure if a list item applies to your research, read the appropriate section before selecting a response.

Materials & experimental systems

n/a	Involved in the study
☐	☒ Antibodies
☐	☒ Eukaryotic cell lines
☒	☐ Palaeontology and archaeology
☒	☐ Animals and other organisms
☒	☐ Human research participants
☒	☐ Clinical data
☒	☐ Dual use research of concern

Methods

n/a	Involved in the study
☒	☐ ChIP-seq
☒	☐ Flow cytometry
☒	☐ MRI-based neuroimaging

Antibodies

Antibodies used	rat monoclonal anti-HA tag antibody conjugated to peroxidase (clone 3F10, Roche, cat# 12 013 819 001)
Validation	Anti-HA-Peroxidase, High Affinity antibody (3F10) recognizes the HA peptide sequence [YPYDVPDYA] derived from the influenza hemagglutinin protein (Kolodziej and Young, 1991, Meth Enzymol 194, 508-511). The Anti-HA-Peroxidase, High Affinity (3F10) antibody is function tested by Western blot analysis using a cell line, that expresses a recombinant HA-tagged protein.

Eukaryotic cell lines

Policy information about cell lines

Cell line source(s)	Cell lines were purchased from the American Type Culture Collection (ATCC). Insect Sf9 cell line: ATCC CRL-1711. Human HEK293 cell line: ATCC-CRL-1573.
Authentication	The cell lines were authenticated by the supplier (ATCC) using morphology and growth characteristics.
Mycoplasma contamination	Cells have been tested and shown to be free from mycoplasma (Hoechst DNA strain and Direct Culture methods employed).

Commonly misidentified lines<br>(See ICLAC register)	No commonly misidentified cell lines were used.
